# Spatiotemporal epidemiology of rabies at an interface between domestic dogs and wildlife in South Africa

**DOI:** 10.1038/s41598-018-29045-x

**Published:** 2018-07-18

**Authors:** Michael Grover, Paul R. Bessell, Anne Conan, Pim Polak, Claude T. Sabeta, Bjorn Reininghaus, Darryn L. Knobel

**Affiliations:** 1 Department of Veterinary Tropical Diseases, University of Pretoria, Pretoria, South Africa; 2 Activating Africa, Hoedspruit, South Africa; 3 The Roslin Institute, The University of Edinburgh, Easter Bush, Edinburgh, United Kingdom; 4 Ross University School of Veterinary Medicine, Basseterre, Saint Kitts and Nevis; 5 Faculty of Veterinary Medicine, Utrecht University, Utrecht, The Netherlands; 6 OIE Rabies Reference Laboratory, Agricultural Research Council - Onderstepoort Veterinary Institute, Pretoria, South Africa; 7 Mpumalanga Veterinary Services, Department of Agriculture, Rural Development, Land and Environmental Affairs, Thulamahashe, South Africa

## Abstract

We characterized the spatiotemporal epidemiology of rabies from January 2009 through March 2014 across the interface between a wildlife reserve and communal livestock farming area in South Africa. Brain tissue from 344 animals of 28 different species were tested for lyssavirus antigen. Of these, 146 (42.4%) samples tested positive, of which 141 (96.6%) came from dogs. Brain samples of dogs were more likely to test positive for lyssavirus antigen if they were found and destroyed in the reserve, compared to samples originating from dogs outside the reserve (65.3% vs. 45.5%; odds ratio (OR) = 2.26, 95% confidence interval (CI) = 1.27–4.03), despite rabies surveillance outside the reserve being targeted to dogs that have a higher index of suspicion due to clinical or epidemiological evidence of infection. In the reserve, dogs were more likely to test positive for rabies if they were shot further from villages (OR = 1.42, 95% CI 1.18–1.71) and closer to water points (OR = 0.41, 95% CI 0.21–0.81). Our results provide a basis for refinement of existing surveillance and control programs to mitigate the threat of spillover of rabies to wildlife populations.

## Introduction

Rabies is an acute, progressive and usually fatal myeloencephalitis that occurs in a wide range of host species. The disease can be caused by infection with a number of related viruses in the genus *Lyssavirus*, of which rabies virus (RABV) is the most widely distributed. While all warm-blooded animals appear susceptible to infection with RABV, relatively few species act as reservoir host, which means being capable of sustaining intraspecific transmission of host-adapted virus strains^1^. Reservoir host species are restricted to the orders Chiroptera (bats) and Carnivora^2^. Non-reservoir hosts (spillover hosts) may be infected following transmission of virus from a reservoir host, but onward transmission in the spillover host population is rare, resulting only in short chains of transmission and extinction of the virus in the spillover population when it does occur^1^. Shifts in the range of reservoir hosts can occur when a RABV variant associated with a particular reservoir species becomes adapted to a spillover host species, such that sustained intraspecies transmission of the new variant occurs in the host population^3^. The inherent biological traits of different species, as well as variations in the population densities within species, appear to be other important determinants of whether certain populations are able to sustain transmission and become reservoir hosts^1^. Among terrestrial hosts capable of serving as reservoirs of RABV, the domestic dog *Canis familiaris* is the most widespread and abundant globally^4^.

Designing effective surveillance and control programs for multi-host pathogens such as RABV can be complex^5^. A useful starting point is to define the species and population (or populations) that the program is intended to benefit. For RABV, these are typically people (public health programs) or livestock (herd health programs), but also include vulnerable or valuable wildlife species (conservation programs), and domestic dogs themselves (animal health and welfare programs). Following Haydon *et al*.^6^, once a target population is identified, the reservoirs of the pathogen of interest can be defined with reference to this population, and control measures can be categorized into those that focus on the target population, the reservoir population(s), or on blocking transmission from reservoirs to targets^7^. The feasibility and cost-effectiveness of different control measures can then be evaluated based on current knowledge. For RABV, these might include creation of herd immunity in the reservoir population through vaccination, restricting interaction between reservoir hosts and target populations through movement control of reservoirs or creation of physical barriers such as fences between populations, or reducing cases in the target population through pre- or post-exposure prophylaxis as well as (in the case of animals) quarantine or euthanasia of exposed individuals.

Surveillance and control of multi-host pathogens present particular challenges in complex socio-ecological systems, such as where people and their domestic animals co-exist in environments with diverse wildlife populations. Transfrontier conservation areas in Africa are large ecological regions that straddle the boundaries of two or more countries, encompassing wildlife protected areas (from which people and their domestic animals are typically excluded) as well as areas of multiple resource use^8^. Effectively addressing the risk of multi-host pathogens in these socio-ecological systems is challenging (see for example Lembo *et al*.^9^) and requires a sound grasp of the epidemiology of the pathogen in the system of interest. For RABV, this includes an understanding of the risk of transmission from infected reservoir hosts to susceptible target hosts, including data on the movement of rabid dogs into wildlife protected areas^10^.

Here, we provide a description of the spatiotemporal epidemiology of rabies in a socio-ecological system with emphasis on its interface between domestic dogs and wildlife, in the eastern part of Mpumalanga Province in South Africa, part of the Greater Limpopo Transfrontier Conservation Area that spans the boundaries of South Africa, Mozambique and Zimbabwe^8^. The target populations of interest were the large predator populations in a private reserve partially surrounded by human settlements with high densities of domestic dogs. After a long absence, rabies reemerged in this area in 2008^11^, posing a threat to the health of wildlife populations in the adjacent reserves, which rely largely on eco-tourism as a source of income. The aim of the study was to characterize the spatiotemporal epidemiology of rabies in the reserve and in a communal livestock farming area to the north of the reserve, especially with regards to incursions of rabid domestic dogs and associated risks for wildlife. Our findings should further improve existing rabies surveillance and control programs, specifically by providing a basis for management decisions to protect large predator populations from this dangerous viral disease.

## Materials and Methods

### Study location

**Figure 1:**
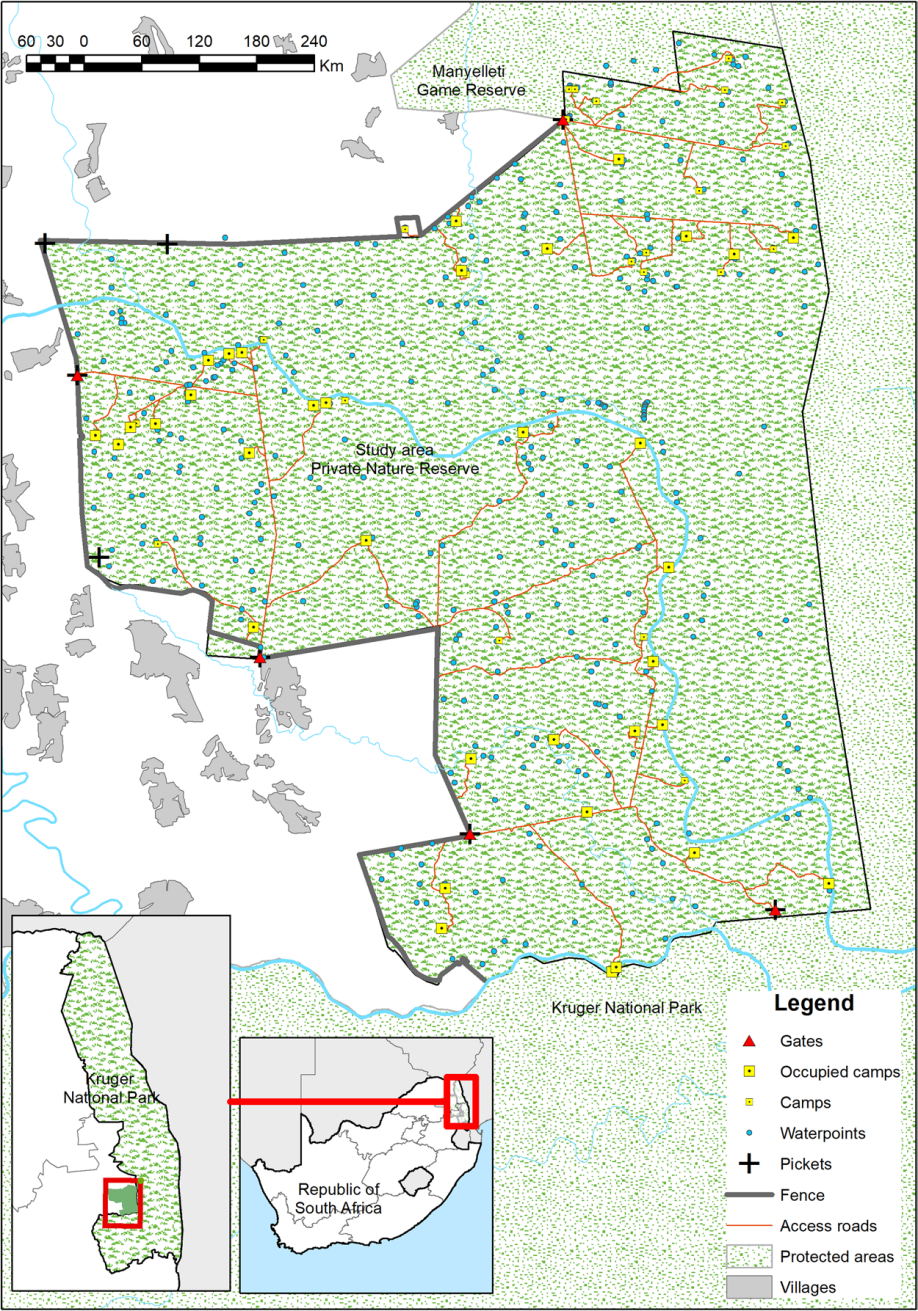
Map of the study reserve indicating the locations and rabies test status of 150 dogs shot from 1 January 2009 through 18 March 2014, along with other relevant spatial features. The map was created using ESRI ArcGIS 10.3 (www.esri.com). Data on protected areas are derived from OpenStreetMap^©^ contributors under a Creative Commons Attribution-ShareAlike 2.0 licence (CC-BY-SA; https://creativecommons.org/licenses/by-sa/2.0/) (made available under the Open Database License: http://opendatacommons.org/licenses/odbl/1.0/. Any rights in individual contents of the database are licensed under the Database Contents License: http://opendatacommons.org/licenses/dbcl/1.0/). Data on administrative boundaries were obtained from the Global Administrative Areas database^22^. Village outlines were digitised by the authors from 1:50,000 topographic maps 2431CA, 2431CB, 2431 CC and 2431CD from the National Geospatial Information of South Africa [Copyright Chief Directorate: National Geospatial Information. Reproduced under Government Printer’s authorisation (Authorisation No. 11793) dated 07 June 2018]. All other data layers were produced and compiled by the authors.

The study reserve (Fig. 1) is a private reserve on the unfenced western boundary of the Kruger National Park (KNP) in eastern Mpumalanga Province, South Africa (24.92°S, 31.47°E). The reserve is part of the administrative area of the Mpumalanga Provincial Veterinary Services (MPVS) State Vet Office: Bushbuckridge East- Orpen (SV BBR E O). The SV BBR E O area also encompasses communal livestock farming areas and other wildlife reserves to the north of the study reserve. Rabies surveillance data for this study were derived from the SV BBR E O office.

The reserve comprises 49,417 ha of privately-owned wildlife-protected area managed by an association and its management team. An additional 13,162 ha of community or state-owned land falls within the boundaries of the protected area, making the total study area 62,579 ha. The reserve shares an unfenced boundary with a Mpumalanga provincial reserve in the north, and the eastern boundary and part of the southern boundaries are open to the KNP. The Sabie River forms most of the southern boundary of the reserve. The total fence line is 70 km long and functions as a veterinary control fence (to contain African buffalo *Syncerus caffer*, the reservoir hosts of the SAT serotypes of foot-and-mouth disease virus). The reserve’s western and northwestern boundary borders community land in Bushbuckridge Local Municipality. There are 27 villages near the reserve, eight of which lie in close proximity to the fence (<2.5 km from the fence line). Two distinct seasons occur in the area: October to March is the warm, wet season and April to September is the cool, dry season.

The fence also restricts movement of small mammals including dogs; however, due to the sandy soils on which the fence is constructed, erosion and digging can make areas vulnerable to incursion of dogs from the outside. At the small non-perennial drainage lines that run under the fence, ground is often soft due to the sandy soil. Additional stakes and fencing are added to these vulnerable areas; however, in heavy rainfall events these can be washed away. Daily fence patrollers close up or fill in any areas that may have been weakened, but after heavy rain falls or periods of less frequent fence patrols, small mammals can sometimes move under the fence using small ditches and gullies. Major flood events can remove longer stretches of fence across and next to larger waterways, which cannot always be closed on the same day. The entire 70 km fence line of the reserve was upgraded over a 23-month period, beginning in January 2010 and ending at the end of November 2011. Fence sections were replaced in 100 m strips at a speed of roughly 8 km of fence replaced per month. No sections of fence were left open overnight and old fence was replaced with new fence to ensure no exit of wildlife or entry of dogs was possible.

### Study design and data collection

This study was conducted retrospectively, using data collected from 1 January 2009 through 18 March 2014 during regular animal disease surveillance and control activities of the local state veterinary services and associated animal control operations of the study reserve. The reserve has best practice management guidelines, catering also for handling intruding domestic dogs and prophylactic disease control measures, especially for conservation purposes. Within this guideline is the authority for reserve management to destroy any stray dogs found on the reserve. General surveillance in the reserve, for security and other purposes, occurs through daily foot patrols on the fence line conducted by field ranger groups. Patrols are frequent and cover the entire extent of the 70 km fence in a 24-hour period. The field rangers are based at four pickets (field ranger outposts) along the western boundary fence as well as at each of the three gates along the fence line. In addition to the foot patrols, two vehicles drive the fence line on a daily basis, each covering a 35 km stretch. Thirty kilometers of fence line are also used as access roads to commercial lodges. Field rangers on foot and in the vehicles report any sightings of stray dogs and may destroy dogs using their standard-issue firearm. Reported dog sightings are followed up by management patrols; dogs are destroyed whenever possible and made available for the local state veterinary services for rabies testing.

An average of 50–70 game drive vehicles traverse the interior roads of the reserve during game drive times, usually 05h00–09h00 and 16h00–19h00 each day. Any sighting of a dog during these drives is reported and again followed up by management using vehicles. A large majority of dogs reported were destroyed, although there were some dogs that could not be located once reported; these are assumed to either have been killed by predators or to have moved back outside the fence line. These numbers were not recorded; however, management estimated it to be less than 5% of the total dogs reported (personal communication, Reserve Management, 2013).

Those tasked with destroying dogs had been trained to do so using a standard-issue rifle and a body placement shot to avoid the head, ensuring the best opportunity for sample collection for rabies diagnosis. Once shot, dog carcasses were collected using gloves, placed into a plastic bag and taken away from the area to a collection point. Reserve management contacted the MPVS for each dog. The State Veterinarian or veterinary technicians of the MPVS (or of the neighbouring Skukuza state veterinary office during some parts of 2009), or in some cases the veterinarian at the nearby Hluvukani Animal Health Clinic, Faculty of Veterinary Science, University of Pretoria, collected brain samples from the obtained dogs. Brain samples, together with test submission forms containing the relevant data, were sent to the Rabies Laboratory at the Onderstepoort Veterinary Institute (OVI), which is a rabies reference laboratory for the World Organisation for Animal Health (OIE). Samples were tested for lyssavirus antigen using the gold-standard direct fluorescent antibody test (FAT)^12^ and results were sent back to the State Veterinarian. Dog carcasses were disposed of by incineration to ashes, or in some instances deep burial.

Field rangers and reserve management collected data on dogs, including time of cull, date, location, sex, as well as any additional notes, and forwarded this to the State Veterinarian or other veterinary official. The respective data was consolidated, supplemented with further relevant info, and captured via the state vet office on the regular rabies test submission forms, as well as in a digital database in Microsoft Excel format, which was made available for data analysis for this study. Data from other animals for rabies sampling, originating from outside of the reserve, was handled by the state veterinary office in the same way. The result of the FAT was matched to the data through the use of the reference codes from the OVI sample registration and the sender, being the local State Vet Office: Bushbuckridge East- Orpen (SV BBR E O). Observation data of any wildlife interactions with dogs were reported and documented immediately by reserve management and distributed to the State Veterinarian. Opportunistic sampling for rabies during routine disease investigation by the local SV office of animal carcasses reported to or found by the reserve management was also conducted in certain instances throughout the period of this retrospective study, using the same methods described above for dogs. Outside the reserve, samples were collected by MPVS as part of their routine animal disease control work. Brain samples from animals for which there was a clinical or epidemiological suspicion of rabies were collected and tested by FAT at the OVI. If more than one dog was destroyed in the same incident, all dogs tested were treated as individual samples, with the exception of nine puppies that were found together in a bag on 15 November 2013; this was treated as one incident as all puppies tested negative and had clearly been placed there by someone.

### Spatial analysis

Data collected without GPS locations was matched using the OVI and state vet’s reference numbers, as well as information from prior reports of field staff, and allocated an approximate GPS point using the descriptions from the report and ArcMap 10.1 (Esri, Redlands, CA). GPS coordinates were converted to decimal degrees as the standard for all of these points. Shapefiles of all georeferenced dogs were created in ArcGIS 10.1 in the WGS84 geographic coordinate system. Existing shapefiles of the following spatial features were collected from reserve management files: (i) camps or lodges in the reserve, (ii) fence line of the reserve, (iii) water points within the reserve, both natural and man-made (excluding rivers but including pans, dams and waterholes which hold water for most of the year), (iv) access roads from gates to camps and lodges, (v) access gates into the reserve, (vi) pickets and staff accommodation, (vii) rivers, classified as minor rivers (rivers that only hold water during high rainfall periods) and major rivers (rivers that hold water almost all year round), (viii) vulnerable points for erosion along the fence line, and (ix) villages bordering the reserve. These shapefiles are ground-truthed shapefiles that originated from the Department of Rural Development and Land Reform’s National Geo-spatial Information from 2006. Using the “Generate near table” tool, run as a batch in the analysis toolbox of Arc Map 10.1, the distance in kilometers was calculated from each record to the nearest feature in each category. All analyses were carried out in the statistical software R, version 2.15.2^13^.

To test for an association between spatial features and rabies test status of dogs in the reserve, spatial data were analysed by logistic regression using a generalized linear model (GLM) with binomial error distribution and *logit* link function in R. Firstly, a univariable analysis of each factor was carried out and variables with p-values < 0.2 were selected for the multivariable GLM. Collinearity between variables was checked using a variance inflation factor (VIF) test in the *car* package^14^ in R. Those factors with VIF scores greater than 5 were removed from the model. All remaining features were included in the multivariable GLM. By removing non-significant terms sequentially in a manual step-wise backward elimination process, the model was reduced to include only variables that were significant at p < 0.05. Interactions between the main effects were then assessed by including an interaction term in the model.

More than half of all dogs destroyed and sampled were located within 200 m of the fence. Results within this distance may be biased by the high surveillance effort along the fence line. A subset analysis was therefore performed to examine the association between spatial features and rabies test results, using only the samples further than 0.2 km from the fence, following the same variable selection and model-building methods above.

### Temporal analysis

For the temporal analysis, records were aggregated by month in which the dog was sampled. Two time series analyses were done on the monthly time series: (1) the number of shot dogs, and (2) the number of rabies positive dogs. A decomposition analysis of the time series data was done to describe the monthly trend and the seasonality of the number of dogs shot, using a decomposition by moving average (function ‘decompose’ in R)^15^. This is an algorithm that was developed to divide up time series into three different components: trend (which describes the long-term progression of the time series), seasonality (reflecting cyclical variation) and random noise. A multiplicative model was used as the seasonality did not appear constant over the five years.

### Ethics

This study was approved by the University of Pretoria Animal Ethics Committee (Protocol No. V002-14). Destruction of domestic dogs is part of the management mandate in the protected area. Testing of clinically or epidemiologically rabies suspect animals is a mandatory work function of the state veterinary services, as part of their regular animal disease control. All methods were performed in accordance with relevant guidelines and regulations.

## Results

During the period from 1 January 2009 through 18 March 2014, 344 animals of 28 different species from the study reserve, adjoining communal farming areas, and other nature reserves within the area of the SV BBR E O office were sampled by and tested through the office for rabies at the OVI. Of these, 146 (42.4%) samples tested positive. Of the 344 samples collected, 236 (68.6%) were from dogs. Of the dogs tested, 141 (59.7%) were positive for rabies. Twelve samples were taken from other domestic animals (eight cats and four cattle) during the study period. Three of the cattle samples (from villages adjacent to the northwest of the reserve), and none of the cat samples tested positive for rabies. Of the 96 samples from wildlife within the SV BBR E O area (including the study reserve and other wildlife reserves), only two tested positive: one from a small spotted genet (*Genetta genetta*) and one from a baboon (*Papio ursinus*). The latter was the only sample from a wild animal in the study reserve that tested positive. This baboon, from a troop known to regularly cross into community land over the fence, was found dead in a camp situated close to the fence.

In the reserve, 170 dogs were tested from 1 January 2009 through 18 March 2014, of which 111 (65.3%) returned a positive FAT result for rabies. A significantly greater proportion of dogs inside the reserve were positive for rabies, compared to those sampled from other areas of SV BBR E O (65.3% vs. 45.5%; Chi-square test with Yates correction, Χ^2^ = 6.978, p = 0.008). The odds of a dog sampled from within the reserve being positive for rabies was 2.26 times greater than for a dog sampled outside the reserve (95% confidence interval CI: 1.27–4.03).

### Spatial analysis

Twenty records for which no location could be established were excluded from the spatial analysis, resulting in a dataset of 150 observations of dogs tested within the reserve. The distribution and rabies test status of these dogs is shown in Fig. 1, together with other relevant spatial features.

Table 1 shows the results of the univariable and multivariable analyses between spatial features and rabies test results of dogs. Distance to water points and distance to villages were associated with rabies test results. The interaction term was not retained in the model. Dogs were more likely to test positive for rabies if they were shot further from villages (odds ratio 1.42, 95% CI 1.18–1.71) and closer to water points (0.41, 95% CI 0.21–0.81).

**Table 1:** Results of univariable and multivariable logistic regression analyses of distance (km) to spatial features and dog rabies test result.

Predictor	Coefficient	Standard error	Odds ratio (95% confidence intervals)
**Univariable results**			
Access roads	−0.375	0.118	0.69 (0.55–0.87)
Camps/lodges	−0.277	0.095	0.76 (0.63–0.92)
Fence	0.519	0.171	1.68 (1.97–2.36)
Gates	−0.021	0.033	0.98 (0.92–1.04)
Major river	−0.129	0.004	0.88 (0.78–0.99)
Minor river	0.187	0.270	1.21 (0.71–2.06)
Pickets	0.249	0.081	1.28 (1.09–1.51)
Villages	0.353	0.093	1.42 (1.18–1.71)
Vulnerable points	0.424	0.145	1.52 (1.15–2.04)
Water points	−0.844	0.316	0.43 (0.23–0.80)
**Multivariable results**			
(Intercept)	0.164	0.375	
Villages	0.351	0.093	1.42 (1.18–1.71)
Water points	−0.892	0.343	0.41 (0.21–0.81)

Table 2 shows the univariable results of the subset analysis, using only those samples further than 0.2 km from the fence (n = 73). The odds ratios of many of the univariable predictors in the subset analysis were similar to the full dataset, with the exception of water points that changed sign, but were not statistically significant. In the final multivariable model, only distance to village remained as a significant factor, with a positive association between distance from a village and a positive rabies test result (odds ratio 1.58, 95% CI 1.13–2.21).

**Table 2:** Results of univariable analyses of distance (km) to spatial features and dog rabies test result, for dogs found more than 0.2 km from the fence.

Predictor	Coefficient	Standard error	Odds ratio (95% confidence intervals)
Access roads	−0.285	0.314	0.75 (0.40–1.41)
Camps/lodges	−0.080	0.225	0.92 (0.59–1.45)
Fence	0.387	0.190	1.47 (1.01–2.15)
Gates	0.051	0.075	1.05 (0.91–1.22)
Major river	−0.176	0.113	0.84 (0.67–1.05)
Minor river	0.499	0.445	1.65 (0.68–4.00)
Pickets	0.201	0.132	1.22 (0.94–1.59)
Villages	0.456	0.169	1.58 (1.13–2.21)
Vulnerable points	0.385	0.191	1.47 (1.01–2.15)
Water points	0.424	0.934	1.53 (0.24–9.83)

### Temporal analysis

**Figure 2:**
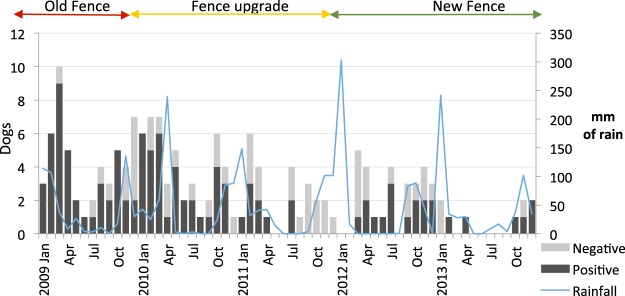
Number of dogs shot by month within the study site showing rabies test results, from 1 January 2009 through 31 December 2013. The blue line and secondary y-axis shows monthly rainfall totals. Indicators at the top of the graph show the status of the fence.

To investigate seasonal trends, data from 1 January 2009 through 31 December 2013 (five calendar years) were used. Nine samples collected from January through March 2014 were excluded, leaving 161 records of dogs tested within the reserve for the temporal analysis. This number is different to that above owing to the inclusion of samples that were missing spatial data. The number of dogs shot by month is shown in Fig. 2. The number of dogs shot decreased over time: 52 dogs were recorded in 2009, 47 in 2010, 25 in 2011, 28 in 2012, and 9 in 2013. The proportions of shot dogs that were rabies positive were 79% in 2009, 74% in 2010, 36% in 2011, 54% in 2012, and 67% in 2013.

To investigate the effect of seasonality (not necessarily related to recorded rainfall), records were categorized into wet (October through March) and dry (April through September) season and analyzed. Of the 161 dogs shot in this period, 105 were shot in the wet season and 56 shot in the dry season. The number of dogs shot in the wet season was significantly higher than in the dry season across the five years of the study (Wilcoxon rank sum test with continuity correction, W = 279, p-value = 0.01).

A fence upgrade took place during the study period, starting in November 2009 and concluding at the end of November 2011. During the old fence period of 11 months, 45 dogs were shot at an average of 4.09 dogs per month (standard deviation [SD] = 2.47, 87% positive for rabies); during the upgrade 78 dogs were shot over 24 months at an average of 3.25 (SD = 2.31, 59% positive); and after the upgrade was completed 47 dogs were shot over 28 months at an average of 1.68 (SD = 1.66, 55% positive for rabies).

**Figure 3:**
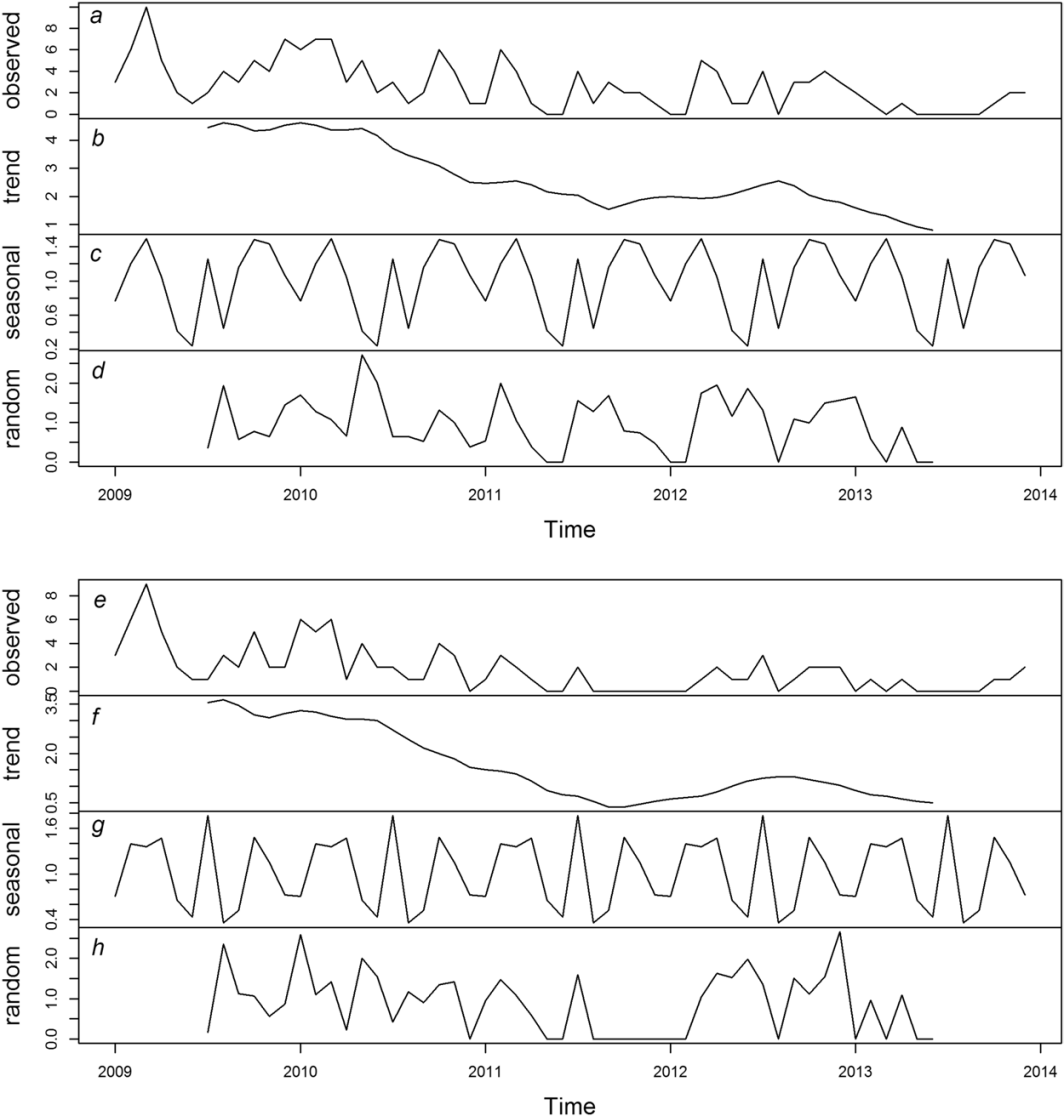
Decomposition of time series for monthly dogs shot (top; **a**–**d**) and rabies positive dogs (bottom; **e**–**h**) from 1 January 2009 through 31 December 2013; (**a**,**e**) observed times series data, (**b**,**f**) generalized trend data over time, (**c**,**g**) modeled seasonal data of time series, and (**d**,**h**) modeled random time series data.

Decomposition analysis of the time series in R was done to separate the trend from the seasonality and is plotted in Fig. 3. The trend data of number of shots dog (Fig. 3b) and number of rabies positive dogs (Fig. 3f) show a general decrease from 2009 through 2013, with a low intensity relapse in 2012. Modeled seasonal data (Fig. 3c,g) indicate a low effect of season compared to the trend and the random data.

## Discussion

Our results are consistent with the theory that domestic dogs were at the time of the study the only reservoir host of RABV in the study area. We found no evidence of maintenance of virus in wildlife in our study area. The large number of rabid dogs found in the reserve does however pose a continual threat to wildlife, as high rates of between-species transmission increase the probability of host shifts occurring.

The substantially higher risk of dogs inside the reserve testing positive for rabies compared to those outside is surprising, as rabies surveillance outside the reserve is targeted to dogs that have a higher index of suspicion due to clinical or epidemiological evidence of infection. Dogs are known to enter the reserve, often accompanied by owners, to chase down small wildlife species. Hunting dogs tend to be of a different phenotype compared with other dogs in the study area, and owners of these dogs are sometimes reluctant to vaccinate their dogs, arguing that it adversely affects their hunting ability (authors’ personal observations). While plausible, a lower vaccination coverage among hunting dogs is unlikely to account for the very high prevalence of rabies in dogs in the reserve, as hunting dogs comprised a relatively small subgroup in some of the studied populations adjacent to the reserve, and overall population immunity was high in these populations, at least during the latter parts of the study period^16^. A more plausible explanation is that sick dogs are deliberately disposed of in the reserve. Options for humane euthanasia of unwanted dogs is limited in the surrounding communities, and litters of unwanted puppies have been found in the reserve. There are anecdotal reports that sick dogs meet a similar fate^17^, but a more detailed anthropological study would be necessary to confirm this theory. A further possibile explanation is that non-rabid dogs in the reserve may evade detection far more successully than rabid dogs.

Dogs in the reserve found further from villages were more likely to be rabid. In a study in Zimbabwe, Butler^18^ found that a majority of rabid dogs (15 out of 24) wandered from their home with the onset of clinical signs, and it is likely that dogs exhibiting atypical, aggressive behaviour would be chased from villages. Although limited, these data suggest that rabid dogs that do wander may not do so aimlessly: a confirmed rabid dog that travelled 11.1 km in one night was found to have followed a route on which it had previously accompanied its owner, before returning home along the same route home^10,18^. Dogs found close to water sources in the reserve were also more likely to be rabid. Hydrophobia, a symptom of rabies in humans, is not noted in dogs^19^, but they do lose the ability to swallow normally due to paralysis of the muscles involved, presumably leading to increased thirst. Rabid cattle, similarly affected, are often found dead near water points^20^, supposedly because they seek relief from fever and thirst. Targeting surveillance around water points may therefore increase detection of rabid dogs in wildife areas.

More stray dogs were detected in the reserve in the wet season compared with the dry season. Fences in the reserve were regularly damaged by flooding drainage lines during high rainfall periods, increasing access points for dogs. The number of dogs recorded in the reserve declined over time, coinciding with the upgrade of the fence. A primary motivation for the fence upgrade was the increase in rhino poaching in the reserve during the study period, and the upgrade was accompanied by other antipoaching measures including increased frequency of patrols and militarization of antipoaching units. It is likely that these security measures would have deterred subsistence poaching of bushmeat by people using hunting dogs, but the degree to which this may have contributed to the decrease in numbers of dogs entering the reserve is not known. The number of rabid dogs detected in the reserve also declined from a peak in 2009. This may reflect the improved coordination of rabies control efforts through mass dog vaccination in the communities surrounding the reserve following the initial reintroduction of the virus in 2008.

This study enhances our understanding of RABV epidemiology at the interface between wildlife conservation reserves and rural farming communities in this area, and provides a basis for refinement of surveillance and control programs to mitigate the threat of spillover to wildlife populations. Maintenance of RABV by a single reservoir host species simplifies approaches, allowing for control measures that focus on that reservoir population^7^. Mechanisms should be created whereby conservation programs can provide logistic or financial support for mass dog vaccination efforts by veterinary services in communities adjacent to reserves, exploiting synergies between public health and conservation goals^21^. Surveillance efforts aimed at detection and removal of rabid dogs from reserves should focus not only at interface areas such as fence lines, but also deeper into reserves further from communities, where in this study a higher proportion of detected dogs were likely to be rabid. This also has implications for protecting target populations of large predators by pre-exposure prophylaxis, for example. The cost-effectiveness of physical barriers such as fences should be evaluated relative to other control options, including rabies education and awareness efforts in communities. Community outreach programs should, where possible, also incorporate results of socio-anthropological studies that address questions around movement of dogs into reserves, disposal of sick and unwanted dogs, and attitudes and practices towards vaccination of dogs among subgroups of owners.
